# Anchoring Issues of CFRP Laminates to Concrete Members

**DOI:** 10.3390/polym14122338

**Published:** 2022-06-09

**Authors:** Justas Slaitas, Remigijus Šalna, Juozas Valivonis

**Affiliations:** Department of Reinforced Concrete Structures and Geotechnics, Vilnius Gediminas Technical University, 10223 Vilnius, Lithuania; remigijus.salna@vilniustech.lt (R.Š.); juozas.valivonis@vilniustech.lt (J.V.)

**Keywords:** carbon fibre reinforced polymer, anchorage, damage, prestressing, bond length, retrofitting

## Abstract

In recent years, carbon fibre reinforced polymer (CFRP) laminates have conquered the structural rehabilitation market due to their ease and quick installation, high strength, anticorrosion properties, and other properties often repeated in the literature. The full potential of these high-strength elements can only be exploited by prestressing. However, the glued laminate joint is partially rigid, resulting in slippage that leads to premature debonding and failure. Therefore, anchoring of the laminate ends is required to stop or delay premature failure and/or perform prestressing. This article discusses the anchoring issues of CFRP laminates and guidelines for the development of anchoring systems. To achieve this goal, the laminate strip was bent, the required clamping forces were determined, possible cases of damage were identified, and individual stress concentrations were modelled. The methodology for calculating the anchor length and the pull-off force is also presented.

## 1. Introduction

The use of carbon fibre reinforced polymer (CFRP) materials for structural rehabilitation has undoubtful benefits. They are light and easy to install (which leads to reduced labour cost), they are thin (aesthetic and design requirements) and more resistant to corrosion compared to steel (durability requirements), although the modulus of elasticity is similar to that of steel [1,2,3,4,5]. It is the modulus of elasticity that makes CFRP a much more suitable reinforcing material than others widely used in construction, such as glass fibre reinforced polymer (GFRP), aramid fibre reinforced polymer (AFRP) [4,6], and basalt fibre reinforced polymer (BFRP) [7,8,9,10,11] with much lower Young’s Modulus.

The modulus of elasticity is particularly important in prestressing, which is a way to exploit the potential of these high-strength materials. The benefits of prestressing include, but are not limited to, the use of full-strength potential, reduced deflection, crack control, improved cracking, and steel reinforcement yield loads of retrofitted structures [3,7,8,9,10,12]. Despite the benefits above, the glued laminate joint is partially rigid, resulting in slippage that leads to premature debonding and failure. Therefore, anchoring of the laminate ends is required to stop or delay premature failure and/or perform prestressing. Studies show that anchoring can increase the bearing capacity of an element by up to 70% compared to strengthening without anchoring the FRP reinforcement [13]. There is a wide variety of different anchors which can be found in the literature [14,15], involving bending the FRP reinforcement, damage to it, and possible eccentricities.

The purpose of this paper is to discuss the possible anchoring issues of particular CFRP laminates and to provide guidelines for the development of anchoring systems. To achieve this goal, the laminate strip was bent, the required clamping forces were determined, and the tests revealed the possible causes of damage when force is applied to bend the laminate. Damage to the laminate decreased its load-bearing capacity by 40% (determined by a direct tensile test). Additionally, to gain a deeper understanding of the problem, different cases of damaged tensile, forced bending, stretching, and eccentrically tensioned CFRP laminate plates were modelled with finite elements. In addition, the methodology for calculating the anchor length and the pulling force is also presented. The proposed numerical technique is based on the theory of built-up bars, which proved to be appropriate for the evaluation of partial shear connection of the retrofitted members [16,17,18,19,20,21,22,23,24]. The four different types of pull-off/out shear tests are used for a comparison of experimental and numerical results: single lap shear, bending shear, double lap shear, and push–pull shear tests [25,26,27,28,29,30,31,32]. The experimental pull-off/out shear test results of concrete blocks strengthened with externally bonded and near-surface mounted carbon fibre (CFRP), glass fibre (GFRP), basalt fibre (BFRP) sheets, laminates, strips, and rods are in a good agreement with numerical results.

## 2. FRP Strengthening Techniques

**Figure 1:**
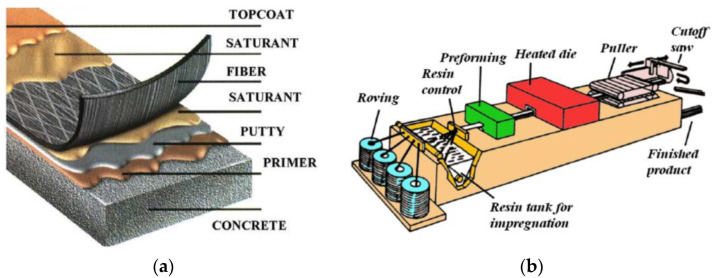
FRP fabrication techniques: (**a**) wet lay-up system. Adapted from Ref. [33]; (**b**) pultrusion process with resin bath impregnation. Adapted from Ref. [34].

The basic FRP strengthening techniques involve the manual application of either wet lay-up or prefabricated systems [6]. The steps to apply the wet lay-up system include (see Figure 1a): grinding the concrete surface, applying primer, smoothing the surface with putty, and applying saturant resin and saturated fibre sheet; the previous step can be repeated with the second and other plies of fibre sheet, applying a top coat.

Prefabricated systems consist of premanufactured cured straight strips, laminates/plates, or rods, which are installed through the use of adhesives. Pre-manufactured FRP reinforcements are usually made through the process of pultrusion (see Figure 1b). This results in much stiffer plastic that cannot be damaged or unfolded, and bending is sufficiently limited compared to a wet lay-up system. Therefore, the use of prefabricated laminates eliminates all anchoring methods in which the laminate needs to be unfolded or damaged in any way.

**Figure 2:**
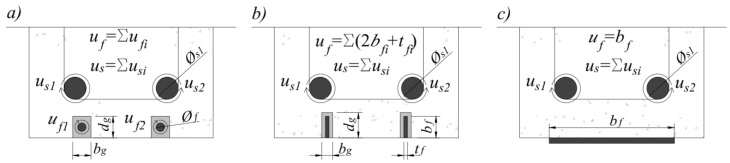
Main types of FRP reinforcements used for strengthening (**a**) NSM rods, (**b**) NSM strips, and (**c**) EBR sheets/laminates.

There are two main FRP systems according to the type of reinforcement and the method of fastening: externally bonded reinforcement (EBR) and near-surface mounted reinforcement (NSM). EBR requires that FRP laminates (pre-manufactured) or sheets (wet lay-up system) be bonded to the external surface of the element (Figure 2c). In the case of NSM, grooves are cut in the concrete surface, into which pre-manufactured FRP strips (Figure 2b) or rods (Figure 2a) are glued.

The problem in laminate folding is also relevant to EBR systems, as folding is not possible in the case of NSM. Therefore, in the next section, the problem of folding/unfolding and eccentric loading of the EBR laminate is discussed.

## 3. Bending and Eccentric Loads of CFRP Laminate

**Figure 3:**
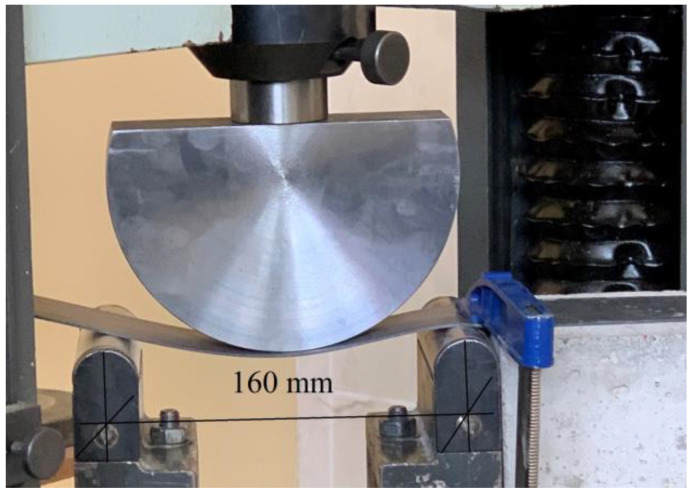
Bending the CFRP laminate with testing machine.

The bending capabilities of the 50 mm-wide CFRP laminate plate (tensile strength *f_f_* = 2627.61 MPa) were first tested. A test machine with a 160 mm base was used for this purpose (Figure 3).

**Figure 4:**
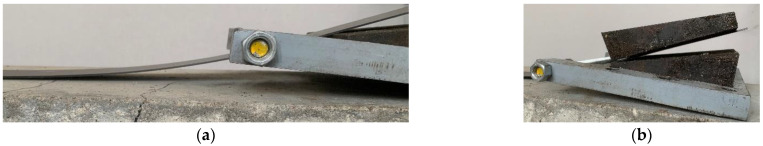

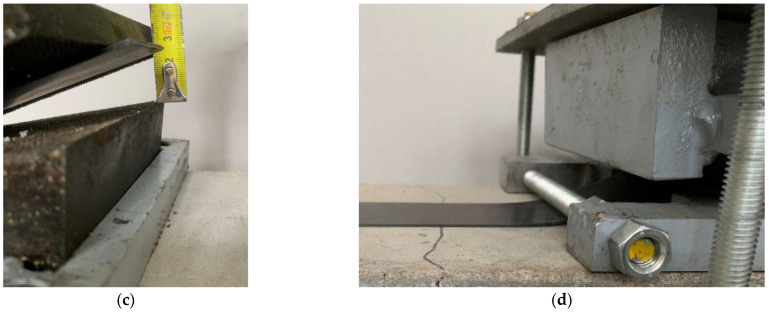
Bending of the CFRP laminate with clamping unit: (**a**) side view front, (**b**) side view back, (**c**) deflection, and (**d**) laminate pressed to concrete.

The maximum bend of the laminate before damage was 25 mm and required a force of 30 kN. Bending the laminate with a clamping unit was tested in the same way, causing the same 25 mm deflection (Figure 4).

**Figure 5:**
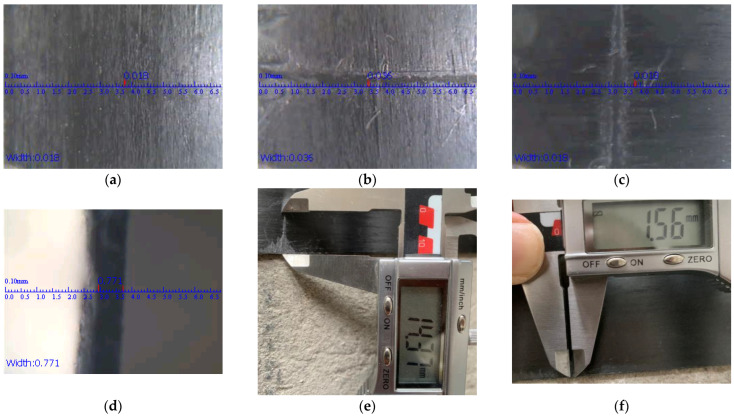
Damage to the laminate: (**a**) undamaged laminate, (**b**) damage image perpendicular to the fibre, (**c**) damage image parallel to the fibre, (**d**) decrease in laminate thickness at the site of damage, (**e**) damage height, and (**f**) damage width.

The test showed that the laminate can be bent up to 25 mm in the anchoring device. However, such forced bending of the laminate damages its structure. Figure 5 shows the damage to the CFRP laminate strip structure that occurred after compression.

**Figure 6:**
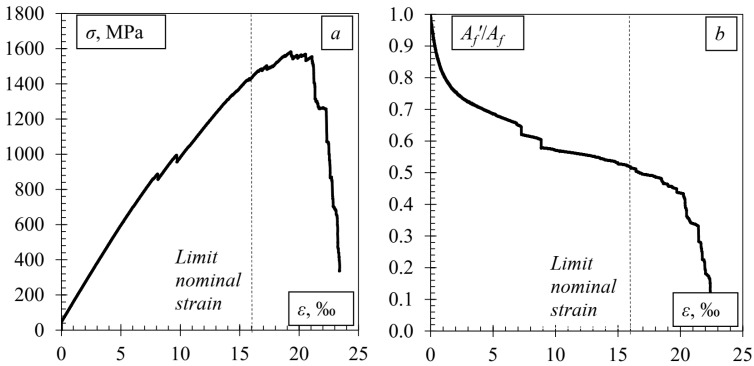
CFRP direct tension test results: (**a**) stress and strain dependence graph; (**b**) reduction in the effective resistance area to the applied force.

The effect of the damage on the bearing capacity of the CFRP tape was determined by direct tension (the test was performed in accordance with [35]). The stress and strain dependence graph is shown in Figure 6.

The damaged CFRP laminate withstands approximately 40% less force than the undamaged one. Furthermore, it should be noted that at the beginning of the load, the laminate was operating in a full cross-sectional area, but as the load increased, the area of the effective laminate decreased until the thickness at the damage site decreased by approximately the same 40%. This is a significant loss of load-bearing capacity and potential of the laminate.

**Figure 7:**
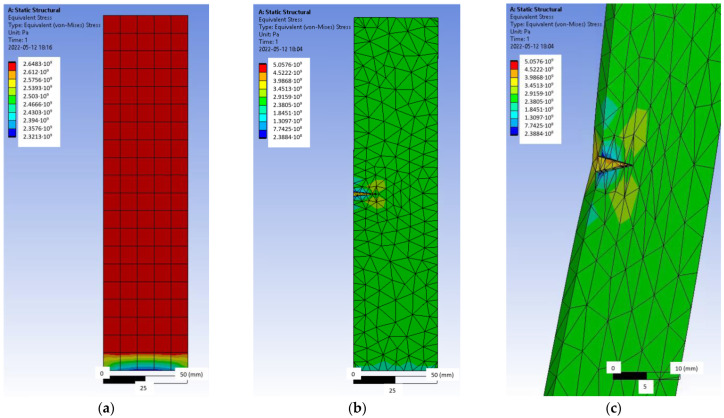
Tensile test simulation with Ansys: (**a**) undamaged laminate, (**b**) damaged laminate, and (**c**) axonometric view of the damaged laminate.

The same tensile test was simulated by the Ansys finite element software (Figure 7). Hexahedral or ‘brick’ finite elements of an average size of 10 mm were used for an undamaged simple geometry plate (Figure 7a), and tetrahedral finite elements were applied to the more complex damaged plate with an adaptive mesh of 1–10 mm (Figure 7b,c). The physical properties of the material used in the analysis are given in Table 1. During the test simulation, one end of the plate was completely restrained, and a force was applied to the free end edge.

As long as the CFRP laminate was intact (Figure 7a), the stresses in it were evenly distributed throughout the strip. However, as damage occurred (Figure 7b,c), the image changed, resulting in a stress concentration at the damage site and at the apex of the crack, resulting in a gradual reduction in the effective area and failure of the laminate.

**Figure 8:**
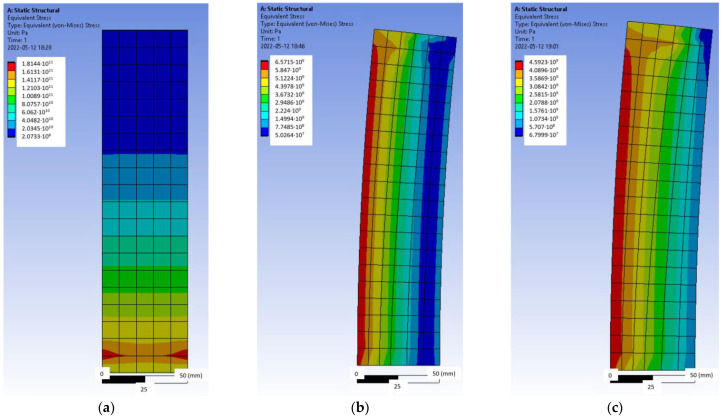
Tensile test simulation with Ansys: (**a**) pressure and tension simultaneously, (**b**) eccentric load action, *e* = 0.25 *b_f_*, and (**c**) eccentric load action, *e* = 0.125 *b_f_*.

The pressure of the CFRP laminate with a force of 30 kN and the tensile force acting together were also simulated, which would correspond to an actual situation (Figure 8a).

Thus, it can be seen from Figure 8a that when pressure and tension work together, a high concentration of stress occurs at the anchoring equipment, which leads to premature failure of the element. Therefore, such an anchoring method with forced bending of the laminate is unacceptable.

The effect of an eccentric force was also simulated (Figure 8b,c). According to previous research conducted at Vilnius Gediminas Technical University, such an eccentric effect can be caused by the tension of the anchor device used for pre-tensioning the laminate instead of pushing. Figure 8b,c shows two cases of eccentricity with high (*e* = 0.25 *b_f_*) and low (*e* = 0.125 *b_f_*) eccentricity; although the stress concentration differs by 1.5 times, in both cases, the eccentricity results in a larger stress difference at the edges of the strip, resulting in the laminate being cut.

**Figure 9:**
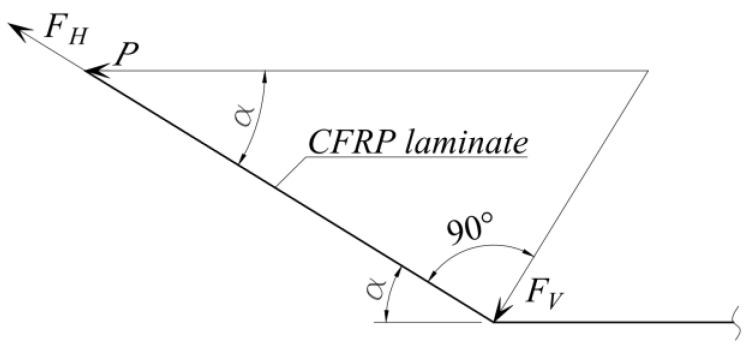
Schematic view of the forces acting on the anchor.

If the eccentricity can be avoided during the prestressing of the laminate by pushing the anchoring device, the bend of the laminate is not avoided, as this greatly increases the efficiency of the anchor. A schematic view of the forces acting on the anchor is shown in Figure 9.

Figure 9 shows the prestressing force *P* and its components at the anchor: the compressing force *F_V_* and the shear force *F_H_* that act specifically on the joint. In this case, it is the load-bearing capacity of the joint:

(1) $$\begin{array}{ccc} T=T_{0}+F_{V}\varphi \geq F_{H}; & F_{V}=P\sin\alpha ; & F_{H}=P\cos\alpha , \end{array}$$

where *T*_0_ is the anchor load capacity, when angle α = 0°, and φ is a coefficient of friction.

**Figure 10:**
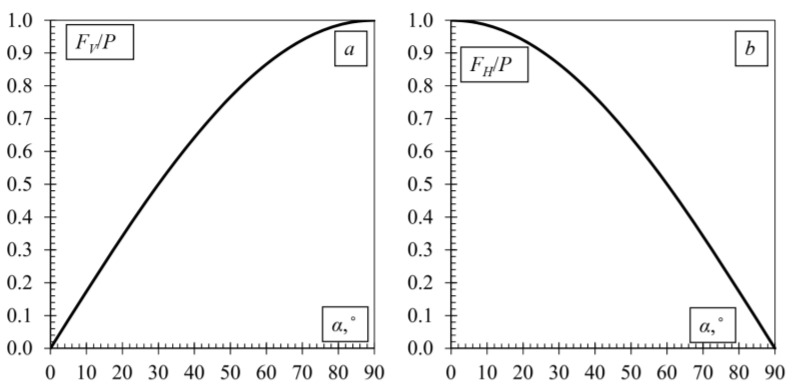
Influence of angle α on tensile force components: (**a**) vertical component; (**b**) horizontal component.

According to Equation (1), the compressive force *F_V_* increases with increasing angle α, while increasing the bearing force of the joint, and at the same time, the force *F_H_* acting on the joint decreases. A schematic representation of this phenomenon is given in Figure 10.

**Figure 11:**
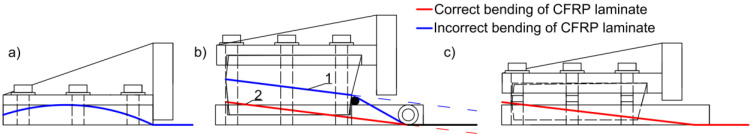
Bending options of CFRP laminate in a clamping unit: (**a**) wavy surface, (**b**) bending with rollers, and (**c**) correct bending of the laminate. Adapted from Ref. [36].

Slaitas and Valivonis [36] found in their proposed prestressing equipment that while using a bending angle α up to 10°, no additional force of pressing the laminate onto the concrete surface is required (Figure 11).

The authors have not been able to find similar studies in the literature, but there are patented prestressing/anchoring devices (e.g., Patent No. CN104895251A) that use wavy surfaces to anchor FRP. The user may be misled by the capabilities of the device. As can be seen from this study, devices with wavy surfaces are not suitable for anchoring prefabricated FRP laminates because, in this case, a force is applied to bend the laminate, which causes stress concentrations, the laminate is damaged, and its load-bearing capacity is significantly reduced. Thus, two different strengthening methods are clearly distinguished at this point; such devices are not suitable for prefabricated FRP laminates, but could be used in wet lay-up strengthening systems.

Another problem observed by the authors that often occurs is the clamping unit in prestressing devices is in tension, while the hydraulic jack is outside of the anchor (e.g., Patent Nos. CN208578344U, CN104895251A, CN1699710A, EP2631392A1). Previous research conducted at Vilnius Gediminas Technical University (Vilnius, Lithuania) has shown that, in this case, it is very difficult to avoid load eccentricities, and very high precision is required (it should be borne in mind that the work should be carried out on a construction site). When even a small eccentricity occurs, a similar result is obtained as in the finite element analysis performed in this article, i.e., a high-stress concentration occurs on one side of the element, and the laminate is cut lengthwise. The research findings, reflections, and guidelines provided should assist scientists and engineers in selecting the appropriate method of strengthening with appropriate anchoring and provide guidance for the development of new anchoring devices.

This section discussed the nature of the anchorage, importance, and possibilities of bending the laminate, the possible eccentricities, and the dangers they pose. However, since FRP laminates are often anchored directly to concrete, for example, by pressing with a metal plate, as was performed in [36], it is no less important to determine the force that the concrete and FRP joint can withstand in the anchor zone (force *T_0_* in Equation (1)) and what the length of the anchor zone itself should be. This is discussed in the next section.

## 4. Load-Bearing Capacity and Anchorage Length of FRP Laminate Anchored Directly to Concrete

The theory of built-up bars was used to describe the length of the anchor. The built-up bars theory has proven to be suitable for this kind of evaluation and extended output, and application examples can be found in the following publications [16,17,18,21,23,37,38,39]. The analytical built-up bars solution of the bond shear force depends on the load conditions of the element. In the case of direct tension/pull-out/pull-off shear tests, there will be no external-bending moment, and the shear stress reaches the exponential shape, similar to that experimentally found in [26,32,40,41,42,43,44,45,46,47,48,49,50]:

(2) $$\tau_{f}\left(z\right)=F_{R}\sqrt{\alpha \beta }e^{-\sqrt{\alpha \beta }z}.$$

When the bond shear force:

(3) $$T_{0}=\int_{0}^{L_{b}}\tau_{f}\left(z\right)dz=F_{R}\left(1-e^{-\sqrt{\alpha \beta }L_{b.eff}}\right).$$

**Figure 12:**
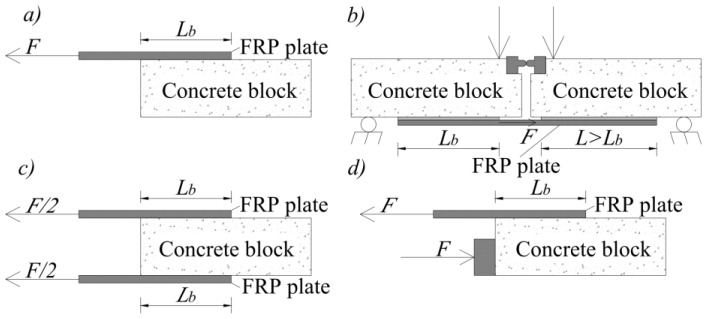
Pull-off/out shear tests (**a**) single lap shear (SLST); (**b**) bending shear (BST); (**c**) double lap shear (DLST); (**d**) push–pull shear (PPST).

The first member of Equation (3) represents the shear force of two rigidly connected members, and the second in brackets is the contact stiffness reduction factor ψ*_f_*:

(4) $$\psi_{f}=1-e^{-\sqrt{\alpha \beta }L_{b.eff}};$$

(5) $$\begin{array}{cc} \alpha =\frac{G_{eff}u_{f}}{h_{ct.eff}}; & \beta =\frac{1}{E_{f}A_{f}}+\frac{1}{E_{cm}A_{c.eff}}+\frac{h_{ct.eff}^{2}}{EI}, \end{array}$$

where *u_f_* is the width of the FRP to the concrete bond (or the perimeter in the case of NSM FRP bars), *E_f_A_f_* is the axial stiffness of the FRP, *E_cm_A_c.eff_* is the axial stiffness of the cracked concrete section, and *EI* is the composite flexural stiffness of two elements (not used for pure tension, PPST, DLST in Figure 12):

(6) $$EI=E_{cm}I_{c.eff}+E_{f}I_{f}+\frac{E_{cm}A_{c.eff}E_{f}A_{f}h_{ct.eff}^{2}}{E_{cm}A_{c.eff}+E_{f}A_{f}},$$

where *E_cm_I_c.eff_* is the flexural stiffness of the cracked concrete section.

The fully analytical solution of the effective shear modulus of concrete and FRP [18]:

(7) $$G_{eff}=\frac{G_{c}t_{a}\left(E_{cm}A_{ct.eff}+E_{f}A_{f}\right)}{L_{b.eff}^{2}u_{f}G_{c}+2h_{ct.eff}\left(E_{cm}A_{ct.eff}+E_{f}A_{f}\right)},$$

where *t_a_* is the thickness of the manufacturer's recommended adhesive layer, the value is between 1 ÷ 4 mm, but for safety reasons, it should be taken equal to *t_a_* = 1 mm; *G_c_* is the shear modulus of the concrete; *A_ct.eff_*—effective area of tensile concrete (Equation (8)) [51]; *L_b.eff_*—effective bond length (e.g., minimum anchorage length) (Equation (9)), which is the lower one: bond length, effective force transfer length [52], concrete crack spacing.

(8) $$A_{ct.eff}=bh_{ct.eff}=\min\left\{ \begin{array}{l} 2.5b\left(h-d_{f}\right);\\ b\left(h-x_{c}\right)/3;\\ bh/2. \end{array}\right.$$

(9) $$L_{b.eff}=\min\left(L_{b},\sqrt{\frac{E_{f}A_{f}}{2f_{ctm}u_{f}}},\frac{2f_{ctm}A_{ct.eff}}{\tau_{fm}u_{f}}\right),$$

where *f_ctm_* is the mean concrete tensile strength and τ*_fm_* is the mean shear stress in the bond, τ*_fm_* ≈ 1.25 *f_ctm_* [6].

It should be noted that the first two members in Equation (8) are valid only for the NSM strengthening technique while talking about pull-out/pull-off shear tests.

The calculation of the force for a fully rigid bond *F_R_* is described below. The equilibrium condition of forces is expressed through FRP strain:

(10) $$\eta \lambda f_{cm}bd_{f}\frac{\varepsilon_{cu}}{\varepsilon_{cu}+\varepsilon_{f}}=A_{f}E_{f}\varepsilon_{f}.$$

FRP strain from Equation (10):

(11) $$\varepsilon_{f}=\frac{\sqrt{{\left(A_{f}E_{f}\varepsilon_{cu}\right)}^{2}+4A_{f}E_{f}\eta \lambda f_{cm}bd_{f}\varepsilon_{cu}}-A_{f}E_{f}\varepsilon_{cu}}{2A_{f}E_{f}}\leq \frac{f_{f}}{E_{f}}.$$

When the depth of the neutral axis:

(12) $$x_{c}=\frac{A_{f}E_{f}\varepsilon_{f}}{\eta \lambda f_{cm}b}.$$

Afterward, the ultimate transferable force through the bond:

(13) $$F_{u}=A_{f}E_{f}\varepsilon_{f}\psi_{f}.$$

In this paper, four types of pull-off/out shear tests were analysed: single lap shear tests (SLST) (Figure 12a), bending shear tests (BST) (Figure 12b), double lap shear tests (DLST) (Figure 12c), and push–pull shear tests (PPST) (Figure 12d).

Different test data covering the test methods discussed above were taken for the pull-off/out shear test verification (see Table 2).

**Figure 13:**
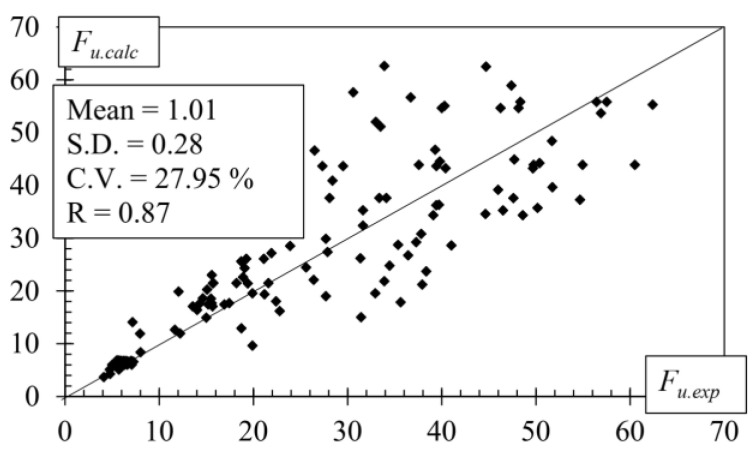
Experimentally calculated pull-off/out ultimate load.

Figure 13 shows the comparison between the ultimate pull-off/out shear forces obtained experimentally and numerically.

A great variety of different pull-off/out shear tests was analysed with a low systematic error (1.01), relatively low random error (0.28) and coefficient of variation (27.95%), and a high coefficient of correlation (0.87). It can be concluded that the ultimate pull-off/out shear load was predicted sufficiently, precisely taking into account the variety of samples. This means that the required anchoring length of the externally bonded and near-surface mounted FRP reinforcement can be determined with sufficient accuracy and ease by varying Equations (3) and (9). 

## 5. Conclusions

(1) The CFRP laminate was bent with a test machine, and it was found that when a force of 30 kN was applied, it could be forcibly bent up to 25 mm; further bending would damage the structure of the specimen. Furthermore, the CFRP laminate with a 25 mm pitch was pressed onto the concrete using a clamping unit. This forced pressing damaged the specimen, the damage depth reached 40% of the CFRP laminate thickness, and the load-bearing capacity, when tested under pure tension, was reduced by a similar amount.
(2) After simultaneously simulating the pressing and tensioning of the laminate with finite element software, the stress concentration was located exactly at the place where the damage occurred in the CFRP laminate, greatly reducing the load-bearing capacity of the element. In view of this, forced bending of the laminate is not recommended.
(3) The effect of eccentric loading can affect the CFRP laminate through the tension of the clamping unit used to prestress the laminate rather than push it. Finite element analysis has shown that eccentricity results in a large stress difference at the edges of the strip, leading to shear failure of the CFRP laminate.
(4) A fully analytical calculation technique is proposed to determine the load-bearing capacity and, at the same time, the anchorage length, based on the theory of built-up bars. The experimental results of the pull-off/pull-out shear test of concrete blocks strengthened with externally bonded and near-surface mounted carbon fibre (CFRP), glass fibre (GFRP), basalt fibre (BFRP) sheets, laminates, strips, and rods are in good agreement with the numerical ones, which shows the great versatility of the proposed method.

## Figures and Tables

**Table 1:** The physical properties of the material used in the analysis.

Property	Value
Density	1800 kg/m^3^
Young’s Modulus x direction	230 GPa
Young’s Modulus y direction	23 GPa
Young’s Modulus z direction	23 GPa
Poisson’s Ratio xy	0.2
Poisson’s Ratio yz	0.4
Poisson’s Ratio xz	0.2
Shear Modulus xy	9 GPa
Shear Modulus yz	8.2 GPa
Shear Modulus xz	9 GPa

**Table 2:** Main parameters of pull-off/out shear tests.

Reference	*A_f_/bd_f_*, %	*L_b_*, mm	*f_cm_*, MPa	*f_f_*, MPa	*E_f_*, GPa	EBR/NSM	Test Type
Bilota et al. [25]	0.05 ÷ 0.53	300	19	1250 ÷ 3194	46 ÷ 221	EBR/NSM	SLST
Sena Cruz and Barros [27]	0.08	40 ÷ 80	33 ÷ 70	2740	158	NSM	BST
Torres et al. [28]	0.17	48 ÷ 240	23	1350 ÷ 2350	64 ÷ 170	NSM	SLST
Novidis et al. [30]	1.24 ÷ 1.27	60 ÷ 120	35	2108	124	NSM	SLST
Costa and Barros [29]	0.06 ÷ 0.12	40 ÷ 300	25 ÷ 41	2833 ÷ 3023	156 ÷ 171	NSM	PPST, DLST, BST
Diab and Farghal [31]	0.17 ÷ 0.5	250	40	2100 ÷ 3400	91 ÷ 230	EBR	DLST
Yao et al. [32]	0.01 ÷ 0.43	75 ÷ 240	19 ÷ 27	351 ÷ 4114	23 ÷ 256	EBR	PPST
Bilota et al. [26]	0.06 ÷ 0.39	300	30 ÷ 42	1208 ÷ 3536	48 ÷ 187	NSM	DLST

Mean values were taken for the same samples.

## Data Availability

All data required to understand and verify the research presented in the article. Data sharing is not applicable to this article.
